# Epigenetic age acceleration is associated with blood lipid levels in a multi-ancestry sample of older U.S. adults

**DOI:** 10.1186/s12920-024-01914-7

**Published:** 2024-05-27

**Authors:** Lisha Lin, Jenna Kiryakos, Farah Ammous, Scott M. Ratliff, Erin B. Ware, Jessica D. Faul, Sharon L.R. Kardia, Wei Zhao, Kira S. Birditt, Jennifer A. Smith

**Affiliations:** 1 Department of Epidemiology, School of Public Health, University of Michigan, 1415 Washington Heights, Ann Arbor, MI 48109 USA; 2 Survey Research Center, Institute for Social Research, University of Michigan, 426 Thompson St, Ann Arbor, MI 48104 USA

**Keywords:** Epigenetic age acceleration, DNA methylation, Lipids, Cholesterol, HDL cholesterol, LDL cholesterol, Triglycerides, Aging

## Abstract

**Background:**

Dyslipidemia, which is characterized by an unfavorable lipid profile, is a key risk factor for cardiovascular disease (CVD). Understanding the relationships between epigenetic aging and lipid levels may help guide early prevention and treatment efforts for dyslipidemia.

**Methods:**

We used weighted linear regression to cross-sectionally investigate the associations between five measures of epigenetic age acceleration estimated from whole blood DNA methylation (HorvathAge Acceleration, HannumAge Acceleration, PhenoAge Acceleration, GrimAge Acceleration, and DunedinPACE) and four blood lipid measures (total cholesterol (TC), LDL-C, HDL-C, and triglycerides (TG)) in 3,813 participants (mean age = 70 years) from the Health and Retirement Study (HRS). As a sensitivity analysis, we examined the same associations in participants who fasted prior to the blood draw (*n* = 2,531) and in participants who did not take lipid-lowering medication (*n* = 1,869). Using interaction models, we also examined whether demographic factors including age, sex, and educational attainment modified the relationships between epigenetic age acceleration and blood lipids.

**Results:**

After adjusting for age, race/ethnicity, sex, fasting status, and lipid-lowering medication use, greater epigenetic age acceleration was associated with lower TC, HDL-C, and LDL-C, and higher TG (*p* < 0.05), although the effect sizes were relatively small (e.g., < 7 mg/dL of TC per standard deviation in epigenetic age acceleration). GrimAge acceleration and DunedinPACE associations with all lipids remained significant after further adjustment for body mass index, smoking status, and educational attainment. These associations were stronger in participants who fasted and who did not use lipid-lowering medication, particularly for LDL-C. We observed the largest number of interactions between DunedinPACE and demographic factors, where the associations with lipids were stronger in younger participants, females, and those with higher educational attainment.

**Conclusion:**

Multiple measures of epigenetic age acceleration are associated with blood lipid levels in older adults. A greater understanding of how these associations differ across demographic groups can help shed light on the relationships between aging and downstream cardiovascular diseases. The inverse associations between epigenetic age and TC and LDL-C could be due to sample limitations or non-linear relationships between age and these lipids, as both TC and LDL-C decrease faster at older ages.

**Supplementary Information:**

The online version contains supplementary material available at 10.1186/s12920-024-01914-7.

## Introduction

Cardiovascular diseases (CVD) are the main causes of morbidity and mortality among U.S. adults [1]. Dyslipidemia, a modifiable major risk factor for CVD [2–4], is characterized by decreased high-density lipoprotein-cholesterol (HDL-C), elevated low-density lipoprotein-cholesterol (LDL-C), and/or elevated triglycerides (TG) [5]. LDL-C makes up the majority of cholesterol in the human body and is associated with a higher risk of CVD [6–8], while higher HDL-C is protective [2, 6, 9]. Elevated TG level is a risk factor for CVD independent of HDL-C levels [10, 11]. Total cholesterol (TC) is the sum of HDL-C, LDL-C, and 20% of TG [12]. In the U.S., approximately 38% of adults have high TC (*≥* 200 mg/dL) [13], and over 60% of adults aged over 65 years have dyslipidemia [14]. Multiple risk factors are associated with the development of dyslipidemia, including behaviors such as unhealthy diet, lack of exercise [15], and genetic factors [16–19]. Epigenetic factors, such as DNA methylation, have also been associated with CVD [20–22], but it is unclear whether changes in the epigenome are precursors or consequences of CVD-related pathologies.

DNA methylation is an epigenetic mechanism that helps regulate transcription without changing the DNA sequence [23]. DNA methylation happens at CpG sites, with the covalent addition of a methyl group to the cytosine base. There has been a recent surge of epigenetic research examining the relationships between biological aging and disease risk or progression [24–26]. Epigenetic clocks are a measure of biological aging assessed via DNA methylation. Epigenetic age acceleration represents the relative difference between one’s chronological age and epigenetic age, where positive values indicate faster biological aging.

Five widely used epigenetic clocks include HorvathAge [27], HannumAge [28], PhenoAge [29], and GrimAge [30], which all assess biological age in years, as well as DunedinPACE, which assesses the pace of aging [31]. Previous studies have found that higher PhenoAge acceleration and DunedinPACE were associated with lower HDL-C and higher TG [29, 32], and higher HannumAge acceleration was associated with lower HDL-C [33, 34] and higher TC [35]. However, the generalization of these findings is unknown because these studies are demographically limited (e.g., comprised of a single race/ethnicity or sex) or did not adjust for important demographic and behavioral factors such as educational attainment and cigarette smoking.

In this study, we examined whether five measures of epigenetic age acceleration were associated with blood lipids and incident CVD in a representative sample of older adults from multiple racial/ethnic groups in the U.S. We also tested whether the associations differed by demographic factors including age, sex, and educational attainment. Characterizing the associations between biological aging and blood lipid levels may help guide the early prevention and treatment efforts for dyslipidemia.

## Methods

### Study sample

The Health and Retirement Study (HRS) is a U.S. nationally representative, longitudinal study of adults aged over 50 years and their spouses from multiple racial/ethnic groups [36]. The study was first conducted in 1992 to collect data on health, financial, and economic factors at both the individual and community levels, with a follow-up every two years. An ancillary study of HRS, the 2016 Venous Blood Study (VBS), collected DNA methylation data and biomarkers such as blood lipid measures, fasting glucose, and inflammatory markers [37]. DNA methylation measurement was performed on a subset of respondents that were randomly selected from the 2016 VBS study. Participants aged 55 and older were assigned a VBS DNA methylation weight, which adjusted for the differential probabilities of participation for age-eligible respondents at the time of the VBS (i.e., those born before 1960) [38, 39]. By incorporating the VBS DNA methylation weight into analysis, the sample is representative of U.S. adults aged 55 years or older. After excluding participants missing all four blood lipid measures (*n* = 10), any of the covariates (*n* = 55), or VBS DNA methylation weight (*n* = 140), a total of 3,813 participants remained in the study. All participants provided written informed consent before participation.

### Lipid measures

Participants were encouraged but not required to fast before the blood draw. TC and TG were both measured in serum using a Roche Cobas 6000 Chemistry Analyzer (Roche Diagnostics Corporation) [40]. Serum HDL-C was measured using the Roche HDL-Cholesterol 3rd generation direct method at Roche Diagnostics (Indianapolis, IN) [40]. LDL-C was only calculated for participants whose TG levels were no higher than 400 mg/dL using Friedewald’s formula: LDL (mg/dL) = TC – HDL-C – (TG/5.0) [41]. HDL-C and TG were natural log-transformed prior to analysis. For each blood lipid measure, values more than 4 standard deviations (SD) from the mean were excluded from the analyses to minimize potential bias due to extreme outliers. This approach was adopted because outlying values could have been due to measurement errors or data entry mistakes. Participants indicated whether they fasted or took lipid-lowering medication prior to the blood draw.

### Incident cardiovascular disease

To capture incident CVD, we used self-reported data obtained from each participant at the 2016 and 2020 study visits from the question, “Has a doctor ever told you that you have had a heart attack, coronary heart disease, angina, congestive heart failure, or other heart problems?”. A total of 2,293 individuals were free of self-reported CVD in 2016. Of those, 205 (8.9%) reported CVD in 2020 and were considered incident cases of CVD.

### DNA methylation and epigenetic age acceleration

#### DNA methylation

DNA methylation was measured in whole blood samples using the Infinium MethylationEPIC BeadChip for 4,104 participants. Samples were randomized across plates by key demographic variables including age, cohort, sex, education, and race/ethnicity [38]. Data preprocessing and quality control were performed using the *minfi* package in the R software. Probes with a detection *P*-value > 0.01 were removed. Samples were removed if they had > 5% missing probes or mismatched sex. The final DNA methylation sample consists of 4,018 participants. Prior to the epigenetic clock estimation, missing beta methylation values were imputed to the mean beta methylation value of the given probe for all samples [38].

#### Epigenetic age acceleration

HorvathAge and HannumAge use CpG sites associated with chronological age. HorvathAge, calculated using 353 CpG sites, was developed in samples of multiple tissues and cell types [27]. HannumAge, calculated using 71 CpG sites, was developed in whole-blood samples [28]. PhenoAge, calculated using 513 CpG sites, was developed to capture chronological aging and 9 aging biomarkers in whole blood samples [29]. GrimAge, which includes 1,030 CpG sites, was developed to capture cigarette smoking pack-years, a known risk for mortality, and other 7 mortality-associated plasma proteins in whole-blood samples [30]. DunedinPACE, a DNA methylation biomarker that uses 173 CpG sites, was developed in blood samples taken longitudinally to capture individual variation in the *rate* of biological aging. All clocks in this study have been publicly released for HRS [38] except for DundeinPACE which we estimated using the DunedinPACE projector provided by Belsky et al. [31].

HorvathAge acceleration (HorvathAA), HannumAge acceleration (HannumAA), PhenoAge acceleration (PhenoAA), GrimAge acceleration (GrimAA), and DunedinPACE were evaluated in this study. All measures of DNA methylation age acceleration (DNAmAA) except for DunedinPACE were estimated by calculating the residuals obtained from regressing each epigenetic age measure on the participants’ chronological age, with a one-unit change representing a one-year increase in biological aging compared to chronological age. The interpretation of DunedinPACE is fundamentally different, with each unit representing a rate of one-year of biological aging per each year of chronological aging. For each measure of DNAmAA, outliers of more than 4 SD from the mean were windsorized to minimize potential bias due to extreme outliers and skewed distributions.

### Covariates

Age at methylation measurement, sex, self-reported race/ethnicity (non-Hispanic White, non-Hispanic Black, Hispanic, all other groups), body mass index (BMI), smoking status (never smoker, former smoker, current smoker), fasting status, lipid-lowering medication use, and educational attainment (less than high school degree, high school degree or equivalent, college degree and above) were collected at the time of the blood draw.

### Statistical analysis

Pearson correlations were calculated among the epigenetic clocks and age acceleration measures, and *P* < 0.05 was considered significant. We next used weighted linear regression to examine the associations between demographic factors including age, sex, educational attainment (3-level variable) as independent variables and each blood lipid measure, adjusting for race/ethnicity, fasting status at blood draw, lipid-lowering medication use, BMI, and smoking status, with *P* < 0.05 considered significant.

We then used weighted linear regression models to explore the associations between each measure of epigenetic age acceleration (independent variable) and each of the blood lipid measures (dependent variable). Model 1 adjusted for age, sex, race/ethnicity, fasting status at blood draw, and lipid-lowering medication use. Model 2 further adjusted for BMI, smoking status, and educational attainment (3-level variable). To allow cross-comparison among the effect sizes of the five epigenetic age acceleration measures, the same associations were examined after standardizing the epigenetic age acceleration measures and lipids. Since five epigenetic age acceleration estimates were tested against four blood lipids, false discovery rate (FDR) was used to correct for multiple testing, and FDR-*P* < 0.05 was considered significant. As a follow-up analysis to better characterize each significant association between epigenetic age acceleration and blood lipids, we investigated the associations between the individual CpGs that comprise the corresponding clock and lipid level. For this exploratory analysis, *P* < 0.05 was considered significant. This analysis was not conducted for GrimAge, as the CpGs that comprise this clock are not publicly available.

Next, we used a similar strategy as above to explore the associations between epigenetic age acceleration and blood lipid measures within each racial/ethnic group separately (Model 2). Since fasting status greatly influences TG [42], and because lipid-lowering medications can have a large impact on lipid profiles as well as DNA methylation [43], we also conducted sensitivity analyses by examining associations (Model 2) after stratifying our sample by fasting status (yes/no) and lipid-lowering medication use (yes/no). Finally, using Model 2, we examined the association between epigenetic age acceleration and incident CVD. For this analysis, *P* < 0.05 was considered significant.

Interactions were only tested for associations in which both the epigenetic age acceleration and the demographic factor were associated with the corresponding blood lipid in Model 2. We introduced multiplicative terms for each demographic factor (age, sex, educational attainment) and epigenetic age acceleration to examine whether the associations differed by these demographic factors. Age was centered for the interaction analyses. To better capture interactions with educational attainment, we examined potential interactions using two dichotomous variables separately: [1] less than high school degree vs. high school degree and above, and [2] less than college degree vs. college degree and above. For significant interactions (*P* < 0.05 for the interaction term), we created plots to illustrate the associations between the epigenetic age acceleration measures and blood lipids at each level of the categorical factors (for sex and educational attainment). For age, we presented the associations at the 25th percentile (62 years old) and 75th percentile (77 years old) of the sample. All statistical analyses were conducted in R software (version 4.3.1) using the *survey*, *emmeans*, and *siPlot* packages.

## Results

The mean age of the participants (*N* = 3,813) was 70.0 years, and the majority were female (57.7%, Table 1). Participants self-identified as non-Hispanic White (67.2%), non-Hispanic Black (16.3%), Hispanic (13.3%), or some other group (3.1%). Over half of the participants had at least a high school degree (59.1%) and 24.3% had at least a college degree. A total of 44.2% were non-smokers, 44.7% were former smokers, and 11.1% were current smokers. The mean BMI was 28.9 kg/m^2^. Nearly half of the participants were using lipid-lowering medications (49.0%), and 66.4% of the participants reported fasting at the time of the blood draw.

**Table 1:** Descriptive characteristics of the Health and Retirement Study participants (*N* = 3,813)

Characteristic	Mean (SD) or *N* (%)	Minimum	Maximum
Age (years)	70.0 (9.3)	56	100
Female sex	2201 (57.7%)		
Race/ethnicity (self-reported)			
Non-Hispanic White	2564 (67.2%)		
Non-Hispanic Black	623 (16.3%)		
Hispanic	509 (13.3%)		
All other groups	117 (3.1%)		
Educational attainment			
Less than high school degree	633 (16.6%)		
High school degree or equivalent	2254 (59.1%)		
College degree and above	926 (24.3%)		
BMI (kg/m^2^)	28.9 (6.3)	14.1	70.7
Smoking status			
Never smoker	1685 (44.2%)		
Former smoker	1704 (44.7%)		
Current smoker	424 (11.1%)		
Lipid-lowering medication use	1869 (49.0%)		
Fasting at time of blood draw	2531 (66.4%)		
**Blood lipids**			
Total cholesterol (mg/dL) (*n* = 3,811)	187.6 (41.6)	79.0	355
HDL-C (mg/dL)^a^	57.0 (18.8)	20.0	159
LDL-C (mg/dL) (*n* = 3,731)	101.7 (35.0)	11.0	239
Triglycerides (mg/dL) (*n* = 3,806)^a^	144.8 (84.0)	33.0	884
**Epigenetic age acceleration**			
HorvathAA (years)	0.017 (6.3)	-25.8	25.9
HannumAA (years)	-0.018 (5.1)	-20.9	20.9
PhenoAA (years)	-0.036 (6.8)	-27.3	27.3
GrimAA (years)	0.009 (4.7)	-17.0	18.9
DunedinPACE	1.038 (0.1)	-32.4	55.0

SD, standard deviation; TC, total cholesterol; HDL-C, high-density lipoprotein; LDL-C, low-density lipoprotein; TG, triglycerides; HorvathAA, HorvathAge acceleration; HannumAA, HannumAge acceleration; PhenoAA, LevineAge (PhenoAge) acceleration; GrimAA, GrimAge acceleration; BMI, body mass index

^a^HDL-C and triglycerides were natural-log transformed prior to analysis

Correlations among the epigenetic clocks ranged from 0.64 to 0.77 (Supplemental Table 1 A), and correlations among DNAmAA measures ranged from − 0.03 to 0.60, with HorvathAA having particularly weak correlations with more recently developed DNAmAA measures (Supplemental Table 1B). All correlations had P-values < 0.05 except for HorvathAA and DunedinPACE.

For the associations between demographic factors and blood lipids in all participants, a 1-year increase in age was associated with a decrease of 0.74 mg/dL, 0.50 mg/dL, and 0.40 mg/dL in TC, LDL-C, and TG at *P* < 0.05 (Supplemental Table 2). Being a female was associated with an increase of 17.87 mg/dL, 14.31 mg/dL, and 6.60 mg/dL in TC, HDL-C, and LDL-C (Supplemental Table 2). Finally, compared with those who did not have a high school degree, having a high school degree or equivalent was associated with an increase of 3.59 mg/dL in HDL-C, and having a college degree and above was associated with a 7.01 mg/dL increase in HDL-C and an 18.29 mg/dL decrease in TG (Supplemental Table 2).

Higher DNAmAA was associated with lower levels of TC, HDL-C, and LDL-C, and higher levels of TG in Model 1 (*P* < 0.05; Table 2) for all DNAmAA measures except for HorvathAA. After further adjustment for BMI, smoking status, and educational attainment (Model 2), GrimAA and DunedinPACE remained associated with all lipid measures. All other associations remained significant although most had slightly attenuated effect estimates, except for PhenoAA with TC and HannumAA with HDL-C, LDL-C and TG, which were no longer significant. Similar patterns were observed when we further examined the standardized epigenetic age acceleration measures and blood lipids (Supplemental Table 3). Among all significant associations in Model 2, DunedinPACE was most strongly associated with all blood lipids, followed by GrimAA. HannumAA and PhenoAA shared similar but smaller estimates. Specifically, a one-unit increase in DunedinPACE was associated with decreases of 38.74 mg/dL, 20.55 mg/dL, and 27.87 mg/dL in TC, HDL-C, and LDL-C, respectively, and an increase of 73.17 mg/dL in TG. Additionally, a one-year increase in GrimAA was associated with decreases of 0.66, 0.51, and 0.62 mg/dL in TC, HDL-C, and LDL-C, respectively, and an increase of 1.75 mg/dL in TG. Because HDL-C and TG were natural log-transformed prior to analyses, these effect size estimates, and those reported subsequently, correspond to a person with the mean blood lipid level in the sample (i.e., HDL-C at 57.0 mg/dL or TG at 144.8 mg/dL). Most of the associations remained significant in non-Hispanic White and non-Hispanic Black samples, with similar effect estimates. Few associations were detected in Hispanics (Supplemental Table 4).

**Table 2:** Associations between epigenetic age acceleration and blood lipids

	Model 1^a^			Model 2^b^			
	β^c^	SE	*P*	β^c^	SE	*P*	FDR-*P*
**TC (** ***n*** ** = 3,811)**							
HorvathAA	0.141	0.109	0.200	0.181	0.110	0.107	0.134
HannumAA	-0.322	0.113	**0.007**	-0.261	0.112	**0.024**	**0.041**
PhenoAA	-0.254	0.105	**0.019**	-0.195	0.103	0.065	0.087
GrimAA	-0.552	0.149	**0.001**	-0.656	0.191	**0.001**	**0.003**
DunedinPACE	-41.90	4.051	**8.35 × 10** ^**− 14**^	-38.74	4.417	**3.95 × 10** ^**− 11**^	**3.95 × 10** ^**− 10**^
**ln(HDL-C) (** ***n*** ** = 3,813)**							
HorvathAA	-0.0001	0.001	0.892	0.001	0.001	0.533	0.533
HannumAA	-0.004	0.001	**0.002**	-0.002	0.001	**0.050**	0.076
PhenoAA	-0.004	0.001	**8.49 × 10** ^**− 5**^	-0.002	0.001	**0.017**	**0.033**
GrimAA	-0.011	0.001	**3.84 × 10** ^**− 10**^	-0.009	0.002	**2.44 × 10** ^**− 5**^	**9.78 × 10** ^**− 5**^
DunedinPACE	-0.633	0.041	**3.56 × 10** ^**− 20**^	-0.447	0.044	**4.08 × 10** ^**− 13**^	**8.15 × 10** ^**− 12**^
**LDL-C (** ***n*** ** = 3,731)**							
HorvathAA	0.069	0.089	0.442	0.084	0.089	0.352	0.391
HannumAA	-0.220	0.106	**0.043**	-0.202	0.106	0.064	0.087
PhenoAA	-0.239	0.091	**0.011**	-0.220	0.090	**0.018**	**0.003**
GrimAA	-0.501	0.142	**0.001**	-0.619	0.166	**0.001**	**0.002**
DunedinPACE	-26.83	4.471	**2.50 × 10** ^**− 7**^	-27.87	4.882	**9.69 × 10** ^**− 7**^	**6.46 × 10** ^**− 6**^
**ln(TG) (** ***n*** **l = 3,806)**							
HorvathAA	0.002	0.002	0.218	0.001	0.002	0.378	0.398
HannumAA	0.004	0.002	**0.042**	0.002	0.002	0.241	0.284
PhenoAA	0.006	0.002	**4.76 × 10** ^**− 4**^	0.004	0.002	**0.012**	**0.027**
GrimAA	0.014	0.002	**2.90 × 10** ^**− 7**^	0.012	0.003	**3.89 × 10** ^**− 5**^	**1.30 × 10** ^**− 4**^
DunedinPACE	0.633	0.064	**4.19 × 10** ^**− 13**^	0.409	0.073	**1.35 × 10** ^**− 6**^	**6.73 × 10** ^**− 6**^

TC, total cholesterol; HDL-C, high-density lipoprotein; LDL-C, low-density lipoprotein; TG, triglycerides; HorvathAA, HorvathAge acceleration; HannumAA, HannumAge acceleration; PhenoAA, PhenoAge acceleration; GrimAA, GrimAge acceleration

^a^Model 1: blood lipid level ~ epigenetic age acceleration + age at methylation measurement + sex + race/ethnicity + fasting status + lipid-lowering medication use

^b^Model 2: Model 1 + body mass index + smoking status + educational attainment (less than high school degree, high school degree or equivalent, college degree and above)

^c^β corresponds to the change in blood lipid level associated with a 1-unit increase in the measure of epigenetic age acceleration

P-value < 0.05 in bold

When we examined the individual CpGs that were included in the clocks, we found that DunedinPACE had the highest percentage of CpGs associated with each lipid except for TC (ranging from 15.0 to 24.3%). For TC, the largest percentage of associated CpGs were from HannumAA (23.9%, Supplemental Table 5). For each lipid, the top 10 significant CpGs are reported, along with annotation information including the corresponding clock(s), chromosomal locations, and nearest gene(s) (Supplemental Tables 6–9). A handful of the top 10 CpGs for each lipid are located within enhancer and promoter regions, and a larger proportion are located at DNAse hypersensitive sites (DHS) which are indicative of active transcription.

We further stratified the sample by fasting status (Supplemental Table 10) and lipid-lowering medication use (Supplemental Table 11), respectively, in Model 2 to see whether the associations between epigenetic age acceleration and lipid levels vary across groups. Associations between DNAmAA and blood lipids were detected in participants who both fasted and did not fast, except for LDL-C, which was only detected in participants who fasted. We also detected associations mainly in participants who did not use lipid-lowering medication at the time of the blood draw. Overall, DunedinPACE had the most consistent associations among the DNAmAA measures, as it was associated with all lipids across all groups except for LDL-C in non-fasting participants.

Finally, a 1-year increase GrimAA was associated with 1.06 higher odds of incident CVD over the next four years (*P* = 0.038; Supplemental Table 12). No other epigenetic age acceleration measures were associated with incident CVD, although DunedinPACE was marginally significant (OR = 3.99; *P* = 0.065).

**Fig. 1:**
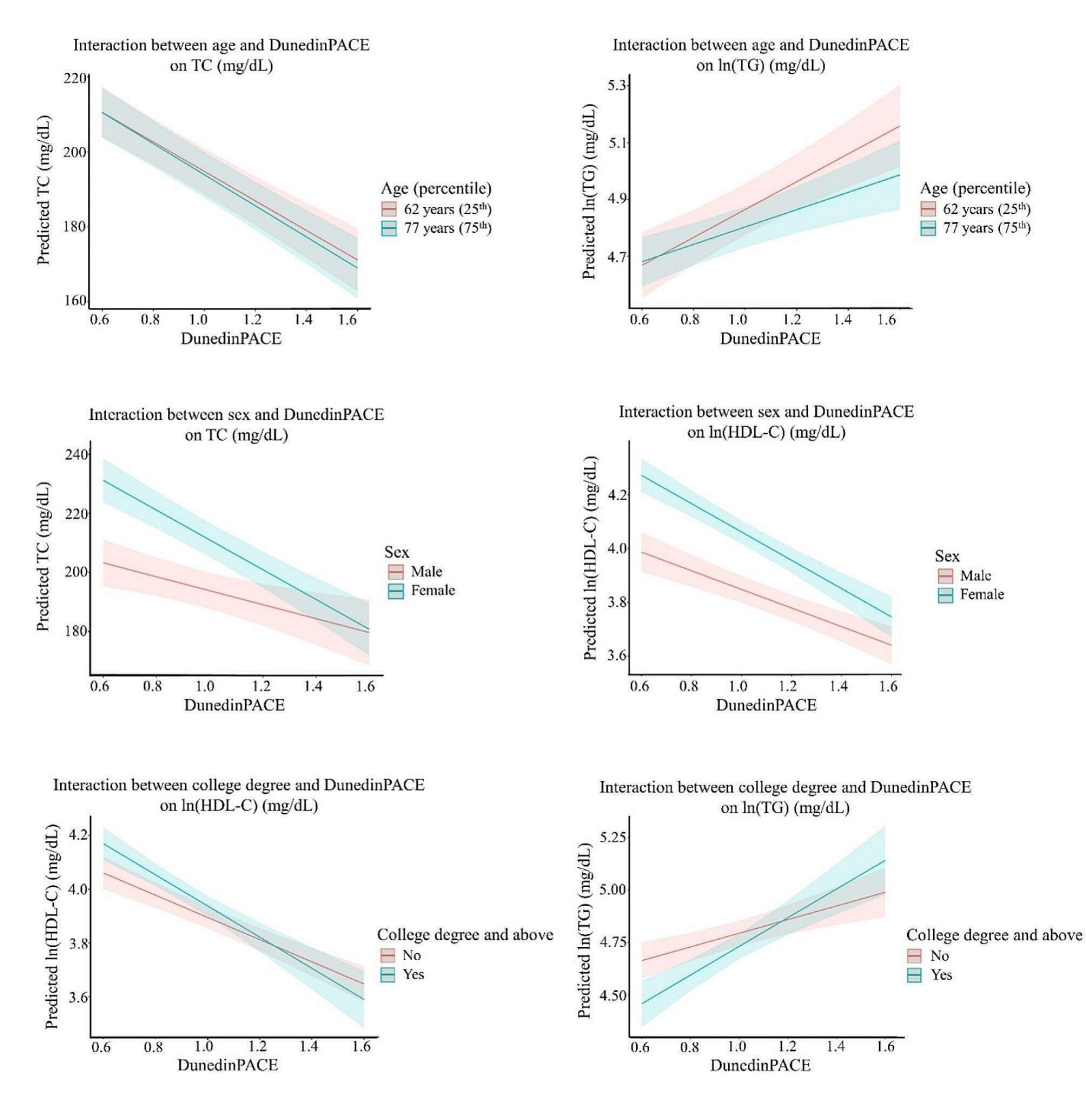
Plots of predicted blood lipid levels by DunedinPACE at the 25th (62 years) and 75th percentile (77 years) of age (top panels), sex (middle panels) and college degree (bottom panels). TC, total cholesterol; HDL-C, high-density lipoprotein; TG, triglycerides Interaction model: blood lipid level ~ epigenetic age acceleration + age at methylation measurement + sex + race/ethnicity + fasting status + lipid-lowering medication use + body mass index + smoking status + high school degree or equivalent + college degree and above + DunedinPACE × demographic factor Only interactions with DunedinPACE and demographic factors with P_interaction_ < 0.05 in the interaction model are shown. Age was centered for the interaction analysis. The line and corresponding confidence intervals represent the predicted blood lipid level at the corresponding value of DunedinPACE

Next, to understand whether associations between DNAmAA and blood lipid levels are modified by demographic factors, we introduced multiplicative terms into Model 2 (Table 3; Fig. 1, Supplemental Fig. 1). Six interactions between DunedinPACE and demographic factors on blood lipids were detected (Fig. 1). For TC, a one-unit increase in DunedinPACE was associated with a decrease of 29.99 mg/dL in younger participants (aged 62 years, 25th percentile) vs. a 49.76 mg/dL in older participants (aged 77 years, 75th percentile) (top left panel, Fig. 1). For TG, a one-unit increase in DunedinPACE was associated with an increase of 42.29 vs. 97.10 mg/dL in younger vs. older participants (top right panel, Fig. 1). In addition, the inverse associations between DunedinPACE and both TC and HDL-C were stronger in females compared with males, as a one-unit increase in DunedinPACE was associated with a decrease of 50.51 vs. 23.65 mg/dL in TC (middle left panel, Fig. 1) and a decrease of 23.32 vs. 16.67 mg/dL in HDL-C in females vs. males (middle right panel, Fig. 1). Finally, for those with a college degree vs. without, a one-unit increase in DunedinPACE was associated with a decrease of 19.29 vs. 25.05 mg/dL in HDL-C (bottom left panel, Fig. 1) and an increase of 55.81 vs. 142.45 mg/dL in TG (bottom right panel, Fig. 1). The significant interactions between GrimAA and PhenoAA and demographic factors on blood lipids showed similar trends (Supplemental Fig. 1.A-C) However, interactions between HannumAA and educational attainment on TG and HDL-C showed different patterns of association than DunedinPACE (Supplemental Fig. 1.D-E).

**Table 3:** Interactions between epigenetic age acceleration and demographic factors on blood lipid levels

Multiplicative term	Lipid	DNAmAA	β_DNAmAA_	*P*_DNAmAA_	β_Demo_	*P*_Demo_	β_Interaction_	*P*_Interaction_
DNAmAA×Age^a^	TC	GrimAA	-0.755	**2.33 × 10** ^**− 4**^	-0.727	**2.51 × 10** ^**− 13**^	-0.051	**0.004**
		DunedinPACE	-40.57	**3.25 × 10** ^**− 12**^	0.760	0.190	-1.318	**0.018**
	ln(TG)	GrimAA	0.011	**4.84 × 10** ^**− 5**^	-0.004	**0.002**	-4.94 × 10^− 4^	**0.037**
		DunedinPACE	0.392	**2.05 × 10** ^**− 6**^	0.008	0.173	-0.012	**0.033**
DNAmAA×Sex^b^	TC	DunedinPACE	-23.65	**0.002**	44.10	**5.62 × 10** ^**− 5**^	-26.86	**0.007**
	ln(HDL-C)	PhenoAA	-0.0005	0.737	0.221	**6.10 × 10** ^**− 23**^	-0.003	**0.037**
		GrimAA	-0.005	**0.033**	0.197	**1.14 × 10** ^**− 19**^	-0.008	**0.007**
		DunedinPACE	-0.346	**8.76 × 10** ^**− 8**^	0.393	**2.34 × 10** ^**− 7**^	-0.180	**0.005**
DNAmAA×College degree^c^	ln(HDL-C)	DunedinPACE	-0.413	**7.81 × 10** ^**− 11**^	0.209	**0.018**	-0.166	**0.049**
	ln(TG)	HannumAA	0.005	0.057	-0.083	**0.002**	-0.008	**0.036**
		DunedinPACE	0.326	**2.46 × 10** ^**− 4**^	-0.422	**0.004**	0.359	**0.011**

TC, total cholesterol; HDL-C, high-density lipoprotein; TG, triglycerides; DNAmAA: epigenetic age acceleration; HannumAA, HannumAge acceleration; PhenoAA, LevineAge (PhenoAge) acceleration; GrimAA, GrimAge acceleration; Demo, demographic factors

Model: blood lipid level ~ epigenetic age acceleration + age at methylation measurement + sex + race/ethnicity + fasting status + lipid-lowering medication use + body mass index + smoking status + high school degree or equivalent + college degree and above + epigenetic age acceleration × demographic factor

We only tested for an interaction when both the DNAmAA and the demographic factor were associated with blood lipids in Model 2

Only significant interactions between epigenetic age acceleration and demographic factors (P_Interaction_<0.05) were included in this table

DNAmAA effect sizes (β_DNAmAA_) correspond to the change in blood lipid level associated with a 1-unit increase in DNAmAA. Demographic factor effect sizes (β_Demo_) correspond to the change in blood lipid level associated with a 1-unit increase in age or with the non-reference level for sex or educational attainment. Interaction effect sizes (β_Interaction_) correspond to the change in effect of β_DNAmAA_ on blood lipids for each 1-unit increase (or level) of the demographic factor

^a^Age was centered in this analysis

^b^Reference group: male

^c^Reference group: less than college degree

P-value < 0.05 in bold

## Discussion

In this study, greater epigenetic age acceleration was associated with lower TC, HDL-C, LDL-C, and higher TG, with stronger associations detected with GrimAA and DunedinPACE. The associations were stronger in participants who fasted prior to blood draw and who did not take lipid-lowering medication, particularly for LDL-C. We detected the largest number of demographic factor interactions with DunedinPACE, in which the associations tended to be stronger among younger participants, females, and those with higher educational attainment. Overall, the effect sizes we observed were relatively small, limiting their potential clinical relevance. However, to our knowledge, this is the first study examining the associations between epigenetic age acceleration and blood lipids, as well as interactions with demographic factors, in a multi-racial/ethnic representative cohort of older U.S. adults.

We found that higher epigenetic age acceleration was associated with lower levels of TC, HDL-C, and LDL-C, as well as higher levels of TG, except for HorvathAA. These results align with previous findings in samples consisting of a single racial/ethnic group. For instance, a 1-year increase in GrimAA was previously associated with an increase of 0.01 log(TG) in older African Americans (mean age = 57 years) [44]. Moreover, a 1-year increase in HannumAA and a 1-SD increase in DunedinPACE were associated with 0.11 mg/dL lower HDL-C and 1.31 times higher odds of hypertriglyceridemia in middle-aged non-Hispanic Whites [33] and Asians [32], respectively.

The inverse associations we detected between epigenetic age acceleration and both TC and LDL-C did not align with our hypothesis that higher epigenetic age would be associated with higher TC and LDL-C, given the adverse effects of these lipids on health [45, 46]. Our finding might be explained by the non-linear relationships between age and TC/LDL-C, as these lipids tend to decrease more rapidly at older ages [47, 48], or the use of lipid-lowering medication among those with high cholesterol. Previous studies also presented similar findings. For example, a 1-year increase in PhenoAA was associated with a decrease of 0.04 mg/dL in TC in older women of multiple race/ethnicities [29]. Also, a 1-year increase in HannumAA was marginally associated with a decrease of 0.17 mg/dL in TC in African Americans [44]. The difference in effect sizes may stem from racial/ethnic differences, age variations, and inclusion criteria in the samples. For example, the average age of our sample (70 years) may have led to selection bias toward healthier participants with lower TC levels. The inverse associations between epigenetic age acceleration and LDL-C we detected were also consistent with previous findings [32, 44].

GrimAA and DunedinPACE exhibited the strongest associations with all blood lipids, while almost no significant associations were detected with earlier developed clocks. Earlier clocks such as HorvathAge and HannumAge were trained on chronological age [27, 28], whereas later clocks including PhenoAge, GrimAge, and DunedinPACE incorporate lifespan mortalities, morbidities, and health behaviors such as smoking [29–31]. GrimAge incorporates CpGs that capture cigarette smoking pack-years, known as an independent risk factor for unfavorable lipid profiles [30, 49]. The stronger associations we observed between GrimAA and lipids could be partially attributed to the residual confounding effects of smoking after we adjusted for smoking as a categorical covariate. DunedinPACE has been associated with social disadvantage and can better predict future functional limitations and chronic disease morbidity than other clocks [31]. Further, when we examined the individual CpGs that comprise the clocks, we found that many of the lipid-associated CpGs in DunedinPACE are located in DNAse hypersensitive sites or near promoters, which are more likely to regulate downstream gene expression and affect health [50–53].

We observed stronger associations, particularly for LDL-C, in participants who fasted prior to the blood draw and those not using lipid-lowering medication. Since TG levels are lower when fasting [42, 54], non-fasting TG measures can lead to underestimation of LDL-C levels when calculated using the Friedewald formula [41]. This could help explain why epigenetic age acceleration was only associated with LDL-C in fasting samples in our study. Similarly, the use of lipid-lowering medications can significantly alter lipid profiles, with notable decreases in TC and LDL-C observed in statin users [43]. The stronger associations between epigenetic age and lipids in participants not using lipid-lowering medication suggests that lipid-lowering medications might mask the relationships.

When we examined the relationship between epigenetic age acceleration and incident CVD, GrimAA was significantly associated and DunedinPACE was marginally associated, both in the expected direction. Previous studies have reported that increased epigenetic age acceleration was associated with higher odds of ischemic stroke [55] and incident coronary heart disease [56]. However, both studies were conducted in participants of European ancestry, and had higher percentages of incident CVD cases (66.7% and 18.4%, respectively) than our analysis (8.9%). Furthermore, the lack of information regarding the specific types of CVD may have contributed to the only marginally significant associations observed in our study.

The stronger inverse association between DunedinPACE and TC in older participants might be explained by faster decreases in TC levels in older adults [47]. On the other hand, the positive association between DunedinPACE and TG was stronger in younger participants, which could be explained by the non-linear relationship of age and TG, with TG levels rising rapidly initially but beginning to decrease in older ages [57, 58]. However, few studies have examined the effects of age on TG levels in longitudinal population studies, limiting our understanding of the associations between epigenetic age acceleration and TG in adults as they grow older.

The associations between DunedinPACE and both TC and HDL-C were negative in both sexes, although stronger in females. The rapid decline in TC and HDL-C levels among females as epigenetic age acceleration increases may be attributed to menopause, given that postmenopausal females had less favorable lipid profiles, including lower TC and HDL-C levels, compared with premenopausal females [59, 60]. We also found that being female was associated with higher levels of HDL-C compared to being male without adjusting for epigenetic age acceleration, which aligns with prior studies [48, 59]. Unhealthy behaviors such as smoking, alcohol consumption, and lack of exercise are associated with dyslipidemia [61], with men being more prone to engaging in these behaviors.

Finally, the associations between higher DunedinPACE and both lower HDL-C and higher TG were both stronger among those with a higher educational attainment. Studies also consistently reported that greater educational attainment and high levels of socioeconomic status are associated with elevated HDL-C levels [62–64], lower prevalence of hypertriglyceridemia [65], and lower epigenetic age [66, 67]. Moreover, epigenetic age acceleration has been inversely associated with HDL-C and positively associated with TG levels [44, 68]. Therefore, the participants in our study who had a college degree might have had healthier HDL-C and TG profiles with lower epigenetic age acceleration, but the effects were attenuated with accelerated biological aging.

This work has many strengths. We were able to examine the associations between epigenetic age acceleration and blood lipids in a nationally representative and multi-racial/ethnic cohort of older U.S. adults. Our success in replicating the results from previous studies consisting of smaller and more homogeneous samples also implies the solid role of biological aging in blood lipid levels across racial/ethnic and demographic groups. In addition, through sensitivity analysis, we were able to examine the associations between epigenetic aging and blood lipids without the influence of fasting status or lipid-lowering medications. Lastly, the interaction analyses shed light on the modification roles of demographic factors on the relationships between epigenetic aging and blood lipids.

Our study also has limitations. First, we cannot infer a causal relationship given that the methylation and blood lipids were measured contemporaneously. Second, the VBS was an optional sub-study for the HRS participants, which could have introduced participation bias. However, by incorporating sample weights, we expect the bias to be minimal. Third, because the epigenome is influenced by the environment, there may be other factors that influenced methylation at the time of collection which cannot be fully evaluated. Fourth, the epigenetic age acceleration measures might not fully capture the methylation changes associated with blood lipids. While the HorvathAge and HannumAge were trained to predict chronological age, the PhenoAge, GrimAge, and DunedinPACE focus on phenotypic traits such as physiological measures, mortality, and the pace of aging. Future studies are warranted to examine the associations between other methylation-based surrogates, such as polyepigenetic scores or lipid-trained epigenetic “clocks”, with lipids in HRS. These endeavors will provide a deeper understanding of the methylation profiles associated with blood lipids. Finally, the effect sizes of the associations we detected were relatively small and thus may provide limited clinical utility for dyslipidemia prevention or treatment. Further, results from this study should be replicated in an independent sample.

## Conclusions

In summary, we have found evidence of associations between epigenetic age acceleration, a powerful biological aging marker, and blood lipid levels, with stronger associations detected with GrimAA and DunedinPACE. Sensitivity analysis highlighted stronger associations in participants who fasted or did not take lipid-lowering medication, particularly for LDL-C. Furthermore, associations between DunedinPACE and blood lipids were stronger in younger participants, females, and individuals with higher educational attainment. The observed association of higher epigenetic age acceleration with lower TC may be influenced by sample limitations or the non-linear relationship between age and TC. Future studies and further exploration of the potential clinical implications of epigenetic age acceleration on blood lipids and other health outcomes are warranted. Overall, our findings contribute to an enhanced understanding of the potential mechanisms linking biological aging and lipid profiles.

### Electronic supplementary material

Below is the link to the electronic supplementary material.

Supplementary Material 1

## Data Availability

HRS epigenetic clocks, blood lipid levels, and survey data are publicly available from the HRS website at https://hrs.isr.umich.edu/data-products. The DunedinPACE clock can be calculated with HRS epigenetic data accessible from the National Institute on Aging Genetics of Alzheimer’s Disease Data Storage Site (NIAGADS), accession number: ng00153, using the codes provided at https://github.com/danbelsky/DunedinPACE/tree/main.

## References

[1] Shah NS, Lloyd-Jones DM, O’Flaherty M, Capewell S, Kershaw KN, Carnethon M (2019). Trends in Cardiometabolic Mortality in the United States, 1999–2017. JAMA.

[2] Nelson RH (2013). Hyperlipidemia as a risk factor for cardiovascular disease. Prim Care.

[3] Karantas ID, Okur ME, Okur NÜ, Siafaka PI (2021). Dyslipidemia management in 2020: an update on diagnosis and therapeutic perspectives. Endocrine, metabolic & Immune disorders-drug targets (formerly current drug targets-Immune. Endocr Metabolic Disorders).

[4] Handelsman Y, Jellinger PS, Guerin CK, Bloomgarden ZT, Brinton EA, Budoff MJ (2020). Consensus statement by the American Association of Clinical Endocrinologists and American College of Endocrinology on the management of dyslipidemia and prevention of cardiovascular disease algorithm–2020 executive summary. Endocr Pract.

[5] Otocka-Kmiecik A, Mikhailidis DP, Nicholls SJ, Davidson M, Rysz J, Banach M (2012). Dysfunctional HDL: a novel important diagnostic and therapeutic target in cardiovascular disease?. Prog Lipid Res.

[6] BM R (1984). The lipid research clinics coronary primary prevention trial results. I. reduction in incidence of coronary heart disease. J Am Med Assoc.

[7] Castelli WP, Garrison RJ, Wilson PW, Abbott RD, Kalousdian S, Kannel WB (1986). Incidence of coronary heart disease and lipoprotein cholesterol levels: the Framingham Study. JAMA.

[8] Castelli WP, Anderson K, Wilson PW, Levy D (1992). Lipids and risk of coronary heart disease the Framingham Study. Ann Epidemiol.

[9] Gordon T, Castelli WP, Hjortland MC, Kannel WB, Dawber TR (1977). High density lipoprotein as a protective factor against coronary heart disease: the Framingham Study. Am J Med.

[10] Sarwar N, Danesh J, Eiriksdottir G, Sigurdsson G, Wareham N, Bingham S (2007). Triglycerides and the risk of coronary heart disease: 10 158 incident cases among 262 525 participants in 29 western prospective studies. Circulation.

[11] Hokanson JE, Austin MA (1996). Plasma triglyceride level is a risk factor for cardiovascular disease independent of high-density lipoprotein cholesterol level: a metaanalysis of population-based prospective studies. J Cardiovasc Risk.

[12] McBride P (2008). Triglycerides and risk for coronary artery disease. Curr Atheroscler Rep.

[13] Virani SS, Alonso A, Aparicio HJ, Benjamin EJ, Bittencourt MS, Callaway CW (2021). Heart Disease and Stroke Statistics-2021 update: a Report from the American Heart Association. Circulation.

[14] McDonald M, Hertz RP, Unger AN, Lustik MB (2009). Prevalence, awareness, and management of hypertension, dyslipidemia, and diabetes among United States adults aged 65 and older. Journals Gerontol Ser A: Biomedical Sci Med Sci.

[15] Franklin BA, Durstine JL, Roberts CK, Barnard RJ (2014). Impact of diet and exercise on lipid management in the modern era. Best Pract Res Clin Endocrinol Metab.

[16] Teslovich TM, Musunuru K, Smith AV, Edmondson AC, Stylianou IM, Koseki M (2010). Biological, clinical and population relevance of 95 loci for blood lipids. Nature.

[17] Asselbergs FW, Guo Y, van Iperen E, Sivapalaratnam S, Tragante V, Lanktree MB (2012). Large-scale gene-centric Meta-analysis across 32 studies identifies multiple lipid loci. Am J Hum Genet.

[18] Discovery (2013). Refinement of loci associated with lipid levels. Nat Genet.

[19] Ripatti P, Rämö JT, Söderlund S, Surakka I, Matikainen N, Pirinen M (2016). The contribution of GWAS loci in familial dyslipidemias. PLoS Genet.

[20] Pearce MS, McConnell JC, Potter C, Barrett LM, Parker L, Mathers JC (2012). Global LINE-1 DNA methylation is associated with blood glycaemic and lipid profiles. Int J Epidemiol.

[21] Nicoletti CF, Nonino CB, de Oliveira BAP, Pinhel MAS, Mansego ML, Milagro FI (2016). DNA methylation and hydroxymethylation levels in relation to two weight loss strategies: energy-restricted diet or bariatric surgery. Obes Surg.

[22] Cash HL, McGarvey ST, Houseman EA, Marsit CJ, Hawley NL, Lambert-Messerlian GM (2011). Cardiovascular disease risk factors and DNA methylation at the LINE-1 repeat region in peripheral blood from Samoan islanders. Epigenetics.

[23] Robertson KD (2005). DNA methylation and human disease. Nat Rev Genet.

[24] Weinhold B. Epigenetics: the science of change. Environ Health Perspect. 1142006. p. A160–7. 10.1289/ehp.114-a160 PMC1392256 16507447

[25] Finkel D, Whitfield K, McGue M (1995). Genetic and environmental influences on functional age: a twin study. Journals Gerontol Ser B: Psychol Sci Social Sci.

[26] Karasik D, Demissie S, Cupples LA, Kiel DP (2005). Disentangling the genetic determinants of human aging: biological age as an alternative to the use of survival measures. Journals Gerontol Ser A: Biol Sci Med Sci.

[27] Horvath S (2013). DNA methylation age of human tissues and cell types. Genome Biol.

[28] Hannum G, Guinney J, Zhao L, Zhang L, Hughes G, Sadda S (2013). Genome-wide methylation profiles reveal quantitative views of human aging rates. Mol Cell.

[29] Levine ME, Lu AT, Quach A, Chen BH, Assimes TL, Bandinelli S (2018). An epigenetic biomarker of aging for lifespan and healthspan. Aging.

[30] Lu AT, Quach A, Wilson JG, Reiner AP, Aviv A, Raj K (2019). DNA methylation GrimAge strongly predicts lifespan and healthspan. Aging.

[31] Belsky DW, Caspi A, Corcoran DL, Sugden K, Poulton R, Arseneault L (2022). DunedinPACE, a DNA methylation biomarker of the pace of aging. Elife.

[32] Lin W-Y (2023). Epigenetic clocks derived from western samples differentially reflect Taiwanese health outcomes. Front Genet.

[33] Irvin MR, Aslibekyan S, Do A, Zhi D, Hidalgo B, Claas SA (2018). Metabolic and inflammatory biomarkers are associated with epigenetic aging acceleration estimates in the GOLDN study. Clin Epigenetics.

[34] Quach A, Levine ME, Tanaka T, Lu AT, Chen BH, Ferrucci L (2017). Epigenetic clock analysis of diet, exercise, education, and lifestyle factors. Aging.

[35] McCartney DL, Stevenson AJ, Walker RM, Gibson J, Morris SW, Campbell A (2018). Investigating the relationship between DNA methylation age acceleration and risk factors for Alzheimer’s disease. Alzheimers Dement (Amst).

[36] Sonnega A, Faul JD, Ofstedal MB, Langa KM, Phillips JW, Weir DR (2014). Cohort profile: the health and retirement study (HRS). Int J Epidemiol.

[37] 2016 Venous Blood Study (VBS). Health and Retirement Study.

[38] Crimmins E, Kim J, Fisher J, Faul J. HRS epigenetic clocks–release 1. Survey Research Center, Univeristy of Michigan; 2020.

[39] Crimmins E, Faul J, Thyagarajan B, Weir D (2017). Venous blood collection and assay protocol in the 2016 Health and Retirement Study.

[40] HRS Documentation Report. Venous blood collection and assay protocol in the 2016 Health and Retirement Study, 2016 venous blood study.: Health and Retirement Study; [.

[41] Friedewald WT, Levy RI, Fredrickson DS (1972). Estimation of the concentration of low-density lipoprotein cholesterol in plasma, without use of the preparative ultracentrifuge. Clin Chem.

[42] Nigam P (2011). Serum lipid profile: fasting or non-fasting?. Indian J Clin Biochem.

[43] Qin X, Wang Y, Pedersen NL, Tang B, Hägg S (2022). Dynamic patterns of blood lipids and DNA methylation in response to statin therapy. Clin Epigenetics.

[44] Ammous F, Zhao W, Ratliff SM, Mosley TH, Bielak LF, Zhou X (2021). Epigenetic age acceleration is associated with cardiometabolic risk factors and clinical cardiovascular disease risk scores in African americans. Clin Epigenetics.

[45] Peters SA, Singhateh Y, Mackay D, Huxley RR, Woodward M (2016). Total cholesterol as a risk factor for coronary heart disease and stroke in women compared with men: a systematic review and meta-analysis. Atherosclerosis.

[46] Nagasawa Sy, Okamura T, Iso H, Tamakoshi A, Yamada M, Watanabe M (2012). Relation between serum total cholesterol Level and Cardiovascular Disease stratified by sex and Age Group: a pooled analysis of 65 594 individuals from 10 Cohort studies in J apan. J Am Heart Association.

[47] Volpato S, Zuliani G, Guralnik JM, Palmieri E, Fellin R (2001). The inverse association between age and cholesterol level among older patients: the role of poor health status. Gerontology.

[48] Feng L, Nian S, Tong Z, Zhu Y, Li Y, Zhang C (2020). Age-related trends in lipid levels: a large-scale cross-sectional study of the general Chinese population. BMJ Open.

[49] Herath P, Wimalasekera S, Amarasekara T, Fernando M, Turale S (2022). Effect of cigarette smoking on smoking biomarkers, blood pressure and blood lipid levels among Sri Lankan male smokers. Postgrad Med J.

[50] Wiench M, John S, Baek S, Johnson TA, Sung MH, Escobar T (2011). DNA methylation status predicts cell type-specific enhancer activity. EMBO J.

[51] Stancheva I, El-Maarri O, Walter J, Niveleau A, Meehan RR (2002). DNA methylation at promoter regions regulates the timing of gene activation in Xenopus laevis embryos. Dev Biol.

[52] Yang M, Park JY. DNA methylation in promoter region as biomarkers in prostate cancer. Cancer Epigenetics: Methods Protocols. 2012:67–109. 10.1007/978-1-61779-612-8_5 PMC3715066 22359288

[53] Tirado-Magallanes R, Rebbani K, Lim R, Pradhan S, Benoukraf T (2017). Whole genome DNA methylation: beyond genes silencing. Oncotarget.

[54] Sidhu D, Naugler C (2012). Fasting time and lipid levels in a community-based population: a cross-sectional study. Arch Intern Med.

[55] Soriano-Tárraga C, Giralt-Steinhauer E, Mola-Caminal M, Vivanco-Hidalgo RM, Ois A, Rodríguez-Campello A (2016). Ischemic stroke patients are biologically older than their chronological age. Aging.

[56] Lind L, Ingelsson E, Sundström J, Siegbahn A, Lampa E (2018). Methylation-based estimated biological age and cardiovascular disease. Eur J Clin Invest.

[57] Jamdar SC, Moon M, Bow S, Fallon HJ (1978). Hepatic lipid metabolism. Age-related changes in triglyceride metabolism. J Lipid Res.

[58] Assmann G, Schulte H (1992). The importance of triglycerides: results from the prospective Cardiovascular Münster (PROCAM) Study. Eur J Epidemiol.

[59] Anagnostis P, Stevenson JC, Crook D, Johnston DG, Godsland IF (2015). Effects of menopause, gender and age on lipids and high-density lipoprotein cholesterol subfractions. Maturitas.

[60] Jensen J, Nilas L, Christiansen C (1990). Influence of menopause on serum lipids and lipoproteins. Maturitas.

[61] Jung C-H, Park J-S, Lee W-Y, Kim S-W. Effects of smoking, alcohol, exercise, level of education, and family history on the metabolic syndrome in Korean adults. Korean J Med. 2002:649–59.

[62] Muennig P, Sohler N, Mahato B (2007). Socioeconomic status as an independent predictor of physiological biomarkers of cardiovascular disease: evidence from NHANES. Prev Med.

[63] Demakakos P, Nazroo J, Breeze E, Marmot M (2008). Socioeconomic status and health: the role of subjective social status. Soc Sci Med.

[64] Benetou V, Chloptsios Y, Zavitsanos X, Karalis D, Naska A, Trichopoulou A (2000). Total cholesterol and HDL-cholesterol in relation to socioeconomic status in a sample of 11,645 Greek adults: the EPIC study in Greece. Scand J Public Health.

[65] Park K-Y, Hong S, Kim K-S, Han K, Park C-Y (2023). Trends in Prevalence of Hypertriglyceridemia and related factors in Korean adults: a serial cross-sectional study. J Lipid Atherosclerosis.

[66] Fiorito G, McCrory C, Robinson O, Carmeli C, Rosales CO, Zhang Y (2019). Socioeconomic position, lifestyle habits and biomarkers of epigenetic aging: a multi-cohort analysis. Aging.

[67] Ryan J, Wrigglesworth J, Loong J, Fransquet PD, Woods RL (2020). A systematic review and meta-analysis of environmental, lifestyle, and health factors associated with DNA methylation age. Journals Gerontology: Ser A.

[68] Huang R-C, Lillycrop KA, Beilin LJ, Godfrey KM, Anderson D, Mori TA (2019). Epigenetic age acceleration in adolescence associates with BMI, inflammation, and risk score for middle age cardiovascular disease. J Clin Endocrinol Metabolism.

