# Supplementary material for: Epigenetic age acceleration is associated with blood lipid levels in a multi-ancestry sample of older U.S. adults
Source: BMC Med Genomics. 2024 May 27;17:146. doi: 10.1186/s12920-024-01914-7 (PMC11129464; doi:10.1186/s12920-024-01914-7)
Supplement: Supplementary file 1 — Supplementary Material 1 [file 12920_2024_1914_MOESM1_ESM.docx]

**Supplemental Table 1A.** Pearson correlation among epigenetic clocks and chronological age

| **Epigenetic clocks** | Age | HorvathAge | HannumAge | PhenoAge | GrimAge |
| --- | --- | --- | --- | --- | --- |
| Age | 1.00 |  |  |  |  |
| HorvathAge | 0.73 | 1.00 |  |  |  |
| HannumAge | 0.81 | 0.77 | 1.00 |  |  |
| PhenoAge | 0.73 | 0.66 | 0.75 | 1.00 |  |
| GrimAge | 0.83 | 0.64 | 0.76 | 0.74 | 1.00 |

All correlations had *P* < 0.05

**Supplemental Table 1B.** Pearson correlation among epigenetic age acceleration measures and chronological age

| **Epigenetic age acceleration** | Age | HorvathAA | HannumAA | PhenoAA | GrimAA | DunedinPACE |
| --- | --- | --- | --- | --- | --- | --- |
| Age | 1.00 |  |  |  |  |  |
| HorvathAA | -0.006 | 1.00 |  |  |  |  |
| HannumAA | -0.003 | 0.43 | 1.00 |  |  |  |
| PhenoAA | 0.006 | 0.28 | 0.42 | 1.00 |  |  |
| GrimAA | -0.001 | 0.10 | 0.26 | 0.35 | 1.00 |  |
| DunedinPACE | 0.104 | -0.03 | 0.21 | 0.38 | 0.60 | 1.00 |

All correlations had *P* < 0.05, except for HorvathAA and DunedinPACE (*P*=0.084).

**Supplemental Table 2**. Associations between demographic factors and blood lipids (N=3,813)

| Blood lipids | TC | | | ln(HDL-C) | | | LDL-C | | | ln(TG) | | |
| --- | --- | --- | --- | --- | --- | --- | --- | --- | --- | --- | --- | --- |
|  | **β^a^** | **SE** | **P** | **β^a^** | **SE** | **P** | **β^a^** | **SE** | **P** | **β^a^** | **SE** | **P** |
| Age | -0.742 | 0.070 | **1.09×10^-13^** | -0.001 | 0.001 | 0.109 | -0.498 | 0.067 | **2.59×10^-9^** | -0.003 | 0.001 | **0.014** |
| Female Sex | 17.874 | 1.247 | **3.42×10^-18^** | 0.224 | 0.011 | **8.84×10^-24^** | 6.600 | 1.138 | **6.66×10^-7^** | -0.022 | 0.017 | 0.191 |
| Educational attainment (ref=less than high school degree) | | | | |  |  |  |  |  |  |  |  |
| High school degree or equivalent | -0.982 | 2.411 | 0.696 | 0.061 | 0.017 | **0.001** | -2.526 | 2.154 | 0.247 | -0.057 | 0.034 | 0.101 |
| College degree and above | -0.318 | 3.103 | 0.919 | 0.116 | 0.021 | **1.38×10^-6^** | -2.061 | 2.967 | 0.491 | -0.135 | 0.038 | **0.001** |

TC, total cholesterol; HDL-C, high-density lipoprotein; LDL-C, low-density lipoprotein; TG, triglycerides

Model: blood lipid level ~ age at methylation measurement + sex + race/ethnicity + fasting status + lipid-lowering medication use + body mass index + smoking status + educational attainment (less than high school, high school degree or equivalent, college degree and above)

^a^β corresponds to the change in blood lipid level associated with a 1-year increase in age, being a female compared with male, having a high school degree or equivalent compared with having less than high school degree, or having a college degree and above compared with having less than high school degree, respectively.

P-value < 0.05 in bold

**Supplemental Table 3**. Associations between standardized epigenetic age acceleration and blood lipids

|  | **Model 1**^a^ | | | **Model 2**^b^ | | |  |
| --- | --- | --- | --- | --- | --- | --- | --- |
|  | **β^c^** | **SE** | **P** | **β^c^** | **SE** | **P** | **FDR-P** |
| **TC (n=3,811)** | |  |  |  |  |  |  |
| HorvathAA | 0.895 | 0.689 | 0.200 | 1.150 | 0.698 | 0.107 | 0.134 |
| HannumAA | -1.644 | 0.579 | **0.007** | -1.334 | 0.572 | **0.024** | **0.041** |
| PhenoAA | -1.722 | 0.710 | **0.019** | -1.322 | 0.698 | 0.065 | 0.087 |
| GrimAA | -2.608 | 0.706 | **0.001** | -3.099 | 0.903 | **0.001** | **0.003** |
| DunedinPACE | -6.119 | 0.592 | **8.35×10^-14^** | -5.658 | 0.645 | **3.95×10^-11^** | **3.95×10^-10^** |
| **ln(HDL-C) (n=3,813)** | |  |  |  |  |  |  |
| HorvathAA | -0.001 | 0.006 | 0.892 | 0.004 | 0.006 | 0.533 | 0.533 |
| HannumAA | -0.021 | 0.007 | **0.002** | -0.012 | 0.006 | **0.050** | 0.076 |
| PhenoAA | -0.026 | 0.006 | **8.49×10^-5^** | -0.016 | 0.006 | **0.017** | **0.033** |
| GrimAA | -0.053 | 0.007 | **3.84×10^-10^** | -0.040 | 0.009 | **2.44×10^-5^** | **9.78×10^-5^** |
| DunedinPACE | -0.093 | 0.006 | **3.56×10^-20^** | -0.065 | 0.006 | **4.08×10^-13^** | **8.15×10^-12^** |
| **LDL-C (n=3,731)** | |  |  |  |  |  |  |
| HorvathAA | 0.438 | 0.565 | 0.442 | 0.532 | 0.566 | 0.352 | 0.391 |
| HannumAA | -1.125 | 0.542 | **0.043** | -1.033 | 0.544 | 0.064 | 0.087 |
| PhenoAA | -1.624 | 0.615 | **0.011** | -1.496 | 0.608 | **0.018** | **0.003** |
| GrimAA | -2.368 | 0.671 | **0.001** | -2.927 | 0.786 | **0.001** | **0.002** |
| DunedinPACE | -3.919 | 0.653 | **2.50×10^-7^** | -4.070 | 0.713 | **9.69×10^-7^** | **6.46×10^-6^** |
| **ln(TG) (n=3,806)** | |  |  |  |  |  |  |
| HorvathAA | 0.014 | 0.011 | 0.218 | 0.009 | 0.010 | 0.378 | 0.398 |
| HannumAA | 0.022 | 0.010 | **0.042** | 0.012 | 0.010 | 0.241 | 0.284 |
| PhenoAA | 0.040 | 0.011 | **4.76×10^-4^** | 0.029 | 0.011 | **0.012** | **0.027** |
| GrimAA | 0.068 | 0.011 | **2.90×10^-7^** | 0.058 | 0.013 | **3.89×10^-5^** | **1.30×10^-4^** |
| DunedinPACE | 0.092 | 0.009 | **4.19×10^-13^** | 0.060 | 0.011 | **1.35×10^-6^** | **6.73×10^-6^** |

TC, total cholesterol; HDL-C, high-density lipoprotein; LDL-C, low-density lipoprotein; TG, triglycerides; HorvathAA, HorvathAge acceleration; HannumAA, HannumAge acceleration; PhenoAA, PhenoAge acceleration; GrimAA, GrimAge acceleration

^a^Model 1: blood lipid level = standardized epigenetic age acceleration + age at methylation measurement + sex + race/ethnicity + fasting status + lipid-lowering medication use

^b^Model 2: Model 1 + body mass index + smoking status + educational attainment (less than high school degree, high school degree or equivalent, college degree and above)

^c^β corresponds to the change in blood lipid level associated with a 1-SD increase in the measure of epigenetic age acceleration.

P-value < 0.05 in bold

**Supplemental Table 4**. Associations between epigenetic age acceleration and blood lipids in the full sample and stratified by racial/ethnic group

|  | **Overall (n=3,813)** | | | **Non-Hispanic White (n=2,563)** | | | **Non-Hispanic Black (n=622)** | | | **Hispanic (n=509)** | | |
| --- | --- | --- | --- | --- | --- | --- | --- | --- | --- | --- | --- | --- |
|  | **β^a^** | **SE** | **P** | **β^a^** | **SE** | **P** | **β^a^** | **SE** | **P** | **β^a^** | **SE** | **P** |
| **TC** | |  |  |  |  |  |  |  |  |  |  |  |
| HorvathAA | 0.181 | 0.110 | 0.107 | 0.141 | 0.127 | 0.275 | -0.157 | 0.243 | 0.521 | 0.094 | 0.299 | 0.757 |
| HannumAA | -0.261 | 0.112 | **0.024** | -0.363 | 0.135 | **0.010** | -0.117 | 0.357 | 0.745 | 0.188 | 0.509 | 0.716 |
| PhenoAA | -0.195 | 0.103 | 0.065 | -0.240 | 0.131 | 0.073 | -0.248 | 0.268 | 0.360 | -0.033 | 0.330 | 0.921 |
| GrimAA | -0.656 | 0.191 | **0.001** | -0.615 | 0.210 | **0.005** | -1.025 | 0.470 | **0.037** | -0.648 | 0.693 | 0.362 |
| DunedinPACE | -38.74 | 4.417 | **3.95×10^-11^** | -35.338 | 4.878 | **4.43×10^-9^** | -29.27 | 11.69 | **0.017** | -55.64 | 18.39 | **0.007** |
| **ln(HDL-C)** | |  |  |  |  |  |  |  |  |  |  |  |
| HorvathAA | 0.001 | 0.001 | 0.533 | 0.001 | 0.001 | 0.256 | -0.001 | 0.002 | 0.439 | -0.001 | 0.002 | 0.450 |
| HannumAA | -0.002 | 0.001 | **0.050** | -0.002 | 0.001 | 0.185 | -0.004 | 0.003 | 0.169 | -0.004 | 0.002 | 0.088 |
| PhenoAA | -0.002 | 0.001 | **0.017** | -0.002 | 0.001 | 0.059 | -0.003 | 0.002 | 0.117 | 4.40×10^-4^ | 0.003 | 0.863 |
| GrimAA | -0.009 | 0.002 | **2.44×10^-5^** | -0.008 | 0.002 | **0.001** | -0.010 | 0.004 | **0.031** | -0.006 | 0.004 | 0.177 |
| DunedinPACE | -0.447 | 0.044 | **4.08×10^-13^** | -0.444 | 0.048 | **6.61×10^-12^** | -0.546 | 0.135 | **3.04×10^-4^** | -0.200 | 0.103 | 0.069 |
| **LDL-C** | |  |  |  |  |  |  |  |  |  |  |  |
| HorvathAA | 0.084 | 0.089 | 0.352 | 0.024 | 0.096 | 0.805 | 0.047 | 0.225 | 0.835 | -0.072 | 0.285 | 0.804 |
| HannumAA | -0.202 | 0.106 | 0.064 | -0.312 | 0.131 | **0.021** | 0.011 | 0.282 | 0.969 | 0.230 | 0.456 | 0.620 |
| PhenoAA | -0.220 | 0.090 | **0.018** | -0.221 | 0.110 | **0.050** | -0.350 | 0.250 | 0.170 | -0.237 | 0.280 | 0.409 |
| GrimAA | -0.619 | 0.166 | **0.001** | -0.568 | 0.188 | **0.004** | -1.062 | 0.312 | **0.002** | -0.716 | 0.579 | 0.232 |
| DunedinPACE | -27.87 | 4.882 | **9.69×10^-7^** | -24.200 | 5.060 | **1.89×10^-5^** | -19.49 | 9.151 | **0.041** | -58.68 | 17.36 | **0.003** |
| **ln(TG)** | |  |  |  |  |  |  |  |  |  |  |  |
| HorvathAA | 0.001 | 0.002 | 0.378 | 0.001 | 0.002 | 0.580 | -0.005 | 0.003 | 0.054 | 0.005 | 0.004 | 0.155 |
| HannumAA | 0.002 | 0.002 | 0.241 | 0.001 | 0.002 | 0.596 | 0.005 | 0.003 | 0.145 | 0.010 | 0.004 | **0.020** |
| PhenoAA | 0.004 | 0.002 | **0.012** | 0.003 | 0.002 | 0.127 | 0.007 | 0.002 | **0.003** | 0.005 | 0.004 | 0.281 |
| GrimAA | 0.012 | 0.003 | **3.89×10^-5^** | 0.010 | 0.003 | **0.006** | 0.018 | 0.006 | **0.004** | 0.009 | 0.007 | 0.249 |
| DunedinPACE | 0.409 | 0.073 | **1.35×10^-6^** | 0.379 | 0.083 | **4.11×10^-5^** | 0.789 | 0.131 | **9.45×10^-7^** | 0.139 | 0.210 | 0.515 |

TC, total cholesterol; HDL-C, high-density lipoprotein; LDL-C, low-density lipoprotein; TG, triglycerides

Model: blood lipid level ~ age at methylation measurement + sex + race/ethnicity + fasting status + lipid-lowering medication use + body mass index + smoking status + educational attainment (less than high school, high school degree or equivalent, college degree and above)

^a^β corresponds to the change in blood lipid level associated with a 1-unit increase in age, being a female compared with male, having a high school degree or equivalent compared with having less than high school degree, or having a college degree and above compared with having less than high school degree, respectively.

P-value < 0.05 in bold

**Supplemental Table 5**. Number (%) of CpGs from each clock associated with each blood lipid level

| **Epigenetic clocks** | **Number of CpGs in the clock** | **Number (%) of CpGs associated with TC at P<0.05** | **Number (%) of CpGs associated with HDL-C at P<0.05** | **Number (%) of CpGs associated with LDL-C at P<0.05** | **Number (%) of CpGs associated with TG at P<0.05** |
| --- | --- | --- | --- | --- | --- |
| HorvathAge | 353 | - | - | - | - |
| HannumAge | 71 | 17 (23.9%) | 14 (19.7%) | - | - |
| PhenoAge | 513 | - | 80 (15.6%) | 58 (11.3%) | 38 (7.4%) |
| DunedinPACE | 173 | 36 (20.8%) | 42 (24.3%) | 26 (15.0%) | 24 (13.9%) |

The association between individual CpGs and blood lipid levels was only examined if the epigenetic age acceleration was significantly associated with the blood lipid measure at FDR-*P* < 0.05.

**Supplemental Table 6.** Top 10 significant associations between CpGs in epigenetic clocks and total cholesterol (TC)

| **CpG** | **Beta** | **SE** | **P** | **Clocks** | **Chr.** | **Pos.** | **UCSC RefGene Name** | **UCSC RefGene Group** | **Relation to UCSC CpG Island** | **Regulatory Feature Group** | **Phantom5 Enhancers^a^** | **DHS^a^** |
| --- | --- | --- | --- | --- | --- | --- | --- | --- | --- | --- | --- | --- |
| cg17901584 | 51.67 | 7.09 | 3.82E-13 | DunedinPACE | chr1 | 55353706 | DHCR24 | TSS1500 | S_Shore | Promoter Associated | 0 | 1 |
| cg09349128 | 94.32 | 16.09 | 4.97E-09 | DunedinPACE | chr22 | 50327986 |  |  | N_Shore | Unclassified | 1 | 1 |
| cg06500161 | -69.76 | 13.50 | 2.47E-07 | DunedinPACE | chr21 | 43656587 | ABCG1 | Body | S_Shore |  | 0 | 0 |
| cg26470501 | 55.56 | 12.14 | 4.90E-06 | DunedinPACE | chr19 | 45252955 | BCL3 | Body | S_Shore | Promoter Associated | 0 | 0 |
| cg14816825 | -59.01 | 13.42 | 1.12E-05 | DunedinPACE | chr11 | 12128203 |  |  | N_Shelf |  | 0 | 0 |
| cg17460386 | -47.93 | 11.11 | 1.66E-05 | DunedinPACE | chr1 | 207095668 | FAIM3 | 5'UTR;  TSS1500 | OpenSea | Unclassified | 0 | 0 |
| cg02650017 | 153.73 | 36.06 | 2.06E-05 | DunedinPACE | chr17 | 47301614 | PHOSPHO1 | Body | Island | Unclassified | 0 | 1 |
| cg06570125 | -60.07 | 15.65 | 0.000126 | DunedinPACE | chr5 | 179723634 |  |  | S_Shelf |  | 0 | 0 |
| ch.13.39564907R | 167.97 | 44.96 | 0.00019 | HannumAge | chr13 | 40666907 |  |  | OpenSea |  | 0 | 0 |
| cg18181703 | 36.27 | 10.67 | 0.000683 | DunedinPACE | chr17 | 76354621 | SOCS3 | Body | N_Shore | Promoter Associated | 0 | 0 |

Chr., chromosome; Pos., position; TSS, transcription start site; UTR, untranslated region; N_Shore, north shore; S_Shore, south shore; UCSC, University of California Santa Cruz Genome Browser; DHS, DNAse hypersensitive site.

^a^ The number “1” indicates that the CpG is located at the corresponding region, whereas “0” indicates not.

**Supplemental Table 7.** Top 10 significant associations between CpGs in epigenetic clocks and high-density lipoprotein (HDL-C)

| **CpG** | **Beta** | **SE** | **P** | **Clocks** | **Chr.** | **Pos.** | **UCSC RefGene Name** | **UCSC RefGene Group** | **Relation to UCSC CpG Island** | **Regulatory Feature Group** | **Phantom5 Enhancers^a^** | **DHS^a^** |
| --- | --- | --- | --- | --- | --- | --- | --- | --- | --- | --- | --- | --- |
| cg06500161 | -1.17 | 0.10 | 1.99E-32 | DunedinPACE | chr21 | 43656587 | ABCG1 | Body | S_Shore |  | 0 | 0 |
| cg17901584 | 0.51 | 0.05 | 1.88E-22 | DunedinPACE | chr1 | 55353706 | DHCR24 | TSS1500 | S_Shore | Promoter Associated | 0 | 1 |
| cg13702222 | -0.39 | 0.06 | 6.61E-10 | DunedinPACE | chr3 | 152017240 | MBNL1 | 1stExon;  5'UTR | OpenSea |  | 0 | 1 |
| cg15551881 | -0.39 | 0.07 | 1.61E-08 | PhenoAge | chr9 | 123688715 | TRAF1 | 5'UTR | N_Shelf | Unclassified | 0 | 1 |
| cg13274938 | -0.52 | 0.10 | 2.29E-07 | DunedinPACE | chr17 | 38493822 | RARA | Body | N_Shelf |  | 0 | 0 |
| cg26470501 | 0.46 | 0.09 | 3.00E-07 | DunedinPACE | chr19 | 45252955 | BCL3 | Body | S_Shore | Promoter Associated | 0 | 0 |
| cg17501210 | 0.33 | 0.07 | 9.52E-07 | DunedinPACE | chr6 | 166970252 | RPS6KA2 | Body | OpenSea |  | 0 | 0 |
| cg11835347 | -0.49 | 0.10 | 1.07E-06 | DunedinPACE | chr1 | 113248232 | RHOC | 5'UTR | N_Shore | Unclassified | 0 | 1 |
| cg18181703 | 0.38 | 0.08 | 1.18E-06 | DunedinPACE | chr17 | 76354621 | SOCS3 | Body | N_Shore | Promoter Associated | 0 | 0 |
| ch.13.39564907R | 1.54 | 0.33 | 2.90E-06 | HannumAge | chr13 | 40666907 |  |  | OpenSea |  | 0 | 0 |

Chr., chromosome; Pos., position; TSS, transcription start site; UTR, untranslated region; N_Shore, north shore; S_Shore, south shore; UCSC, University of California Santa Cruz Genome Browser; DHS, DNAse hypersensitive site.

^a^ The number “1” indicates that the CpG is located at the corresponding region, whereas “0” indicates not.

**Supplemental Table 8.** Top 10 significant associations between CpGs in epigenetic clocks and low-density lipoprotein (LDL-C)

| **CpG** | **Beta** | **SE** | **P** | **Clocks** | **Chr.** | **Pos.** | **UCSC RefGene Name** | **UCSC RefGene Group** | **Relation to UCSC CpG Island** | **Regulatory Feature Group** | **Phantom5 Enhancers^a^** | **DHS^a^** |
| --- | --- | --- | --- | --- | --- | --- | --- | --- | --- | --- | --- | --- |
| cg06500161 | -71.20 | 11.77 | 1.62E-09 | DunedinPACE | chr21 | 43656587 | ABCG1 | Body | S_Shore |  | 0 | 0 |
| cg25536676 | 95.00 | 16.99 | 2.40E-08 | PhenoAge | chr1 | 55353327 | DHCR24 | TSS1500 | Island |  | 0 | 1 |
| cg17901584 | 33.58 | 6.15 | 4.99E-08 | DunedinPACE | chr1 | 55353706 | DHCR24 | TSS1500 | S_Shore | Promoter Associated | 0 | 1 |
| cg21120249 | 44.42 | 10.74 | 3.60E-05 | PhenoAge | chr9 | 139921971 | C9orf139;  ABCA2 | 5'UTR;  1stExon;  Body | N_Shore | Promoter Associated | 0 | 0 |
| cg13718960 | -75.32 | 18.75 | 6.02E-05 | PhenoAge | chr14 | 21271313 | RNASE1 | TSS1500 | OpenSea |  | 0 | 0 |
| cg09349128 | 54.59 | 13.97 | 9.45E-05 | DunedinPACE | chr22 | 50327986 |  |  | N_Shore | Unclassified | 0 | 1 |
| cg26470501 | 40.93 | 10.51 | 0.000101 | DunedinPACE | chr19 | 45252955 | BCL3 | Body | S_Shore | Promoter Associated | 0 | 0 |
| cg05125838 | 62.72 | 16.30 | 0.00012 | PhenoAge | chr3 | 48601585 | UCN2;  COL7A1 | TSS1500;3'UTR | OpenSea |  | 0 | 1 |
| cg14816825 | -44.10 | 11.66 | 0.000158 | DunedinPACE | chr11 | 12128203 |  |  | N_Shelf |  | 0 | 0 |
| cg23668631 | 55.20 | 15.12 | 0.000265 | PhenoAge | chr17 | 3796936 | CAMKK1 | TSS1500 | S_Shore |  | 0 | 0 |

Chr., chromosome; Pos., position; TSS, transcription start site; UTR, untranslated region; N_Shore, north shore; S_Shore, south shore; UCSC, University of California Santa Cruz Genome Browser; DHS, DNAse hypersensitive site.

^a^ The number “1” indicates that the CpG is located at the corresponding region, whereas “0” indicates not.

**Supplemental Table 9.** Top 10 significant associations between CpGs in epigenetic clocks and Triglycerides (TG)

| **CpG** | **Beta** | **SE** | **P** | **Clocks** | **Chr.** | **Pos.** | **UCSC RefGene Name** | **UCSC RefGene Group** | **Relation to UCSC CpG Island** | **Regulatory Feature Group** | **Phantom5 Enhancers^a^** | **DHS^a^** |
| --- | --- | --- | --- | --- | --- | --- | --- | --- | --- | --- | --- | --- |
| cg06500161 | 2.12 | 0.16 | 1.29E-37 | DunedinPACE | chr21 | 43656587 | ABCG1 | Body | S_Shore |  | 0 | 0 |
| cg00574958 | -3.50 | 0.40 | 7.16E-18 | DunedinPACE | chr11 | 68607622 | CPT1A | 5'UTR | N_Shore |  | 0 | 1 |
| cg06690548 | -0.76 | 0.13 | 2.54E-09 | PhenoAge | chr4 | 139162808 | SLC7A11 | Body | OpenSea |  | 0 | 1 |
| cg17901584 | -0.45 | 0.09 | 2.53E-07 | DunedinPACE | chr1 | 55353706 | DHCR24 | TSS1500 | S_Shore | Promoter Associated | 0 | 1 |
| cg18513344 | -1.15 | 0.23 | 8.19E-07 | DunedinPACE | chr3 | 195531298 | MUC4 | Body | OpenSea | Unclassified | 0 | 1 |
| cg13274938 | 0.79 | 0.17 | 2.64E-06 | DunedinPACE | chr17 | 38493822 | RARA | Body | N_Shelf |  | 0 | 0 |
| cg11835347 | 0.72 | 0.17 | 2.01E-05 | DunedinPACE | chr1 | 113248232 | RHOC | 5'UTR | N_Shore | Unclassified | 0 | 1 |
| cg17061862 | -0.38 | 0.09 | 6.24E-05 | DunedinPACE | chr11 | 9590431 |  |  | N_Shelf |  | 1 | 1 |
| cg13702222 | 0.39 | 0.11 | 2.75E-04 | DunedinPACE | chr3 | 152017240 | MBNL1 | 1stExon;5'UTR | OpenSea |  | 0 | 1 |
| cg12547807 | -0.56 | 0.16 | 4.84E-04 | DunedinPACE | chr1 | 9473751 |  |  | OpenSea | Unclassified | 1 | 1 |

Chr., chromosome; Pos., position; TSS, transcription start site; UTR, untranslated region; N_Shore, north shore; S_Shore, south shore; UCSC, University of California Santa Cruz Genome Browser; DHS, DNAse hypersensitive site.

^a^ The number “1” indicates that the CpG is located at the corresponding region, whereas “0” indicates not.

**Supplemental Table 10.** Associations between epigenetic age acceleration and total cholesterol or LDL-C levels in participants by fasting status at the time of cholesterol measurement

|  | Fasting^a^ | | Non-fasting^b^ | |
| --- | --- | --- | --- | --- |
|  | **β^c^** | **P** | **β^c^** | **P** |
| **TC** |  |  |  |  |
| HorvathAA | 0.318 | **0.025** | -0.138 | 0.402 |
| HannumAA | -0.213 | 0.113 | -0.391 | 0.154 |
| PhenoAA | -0.228 | 0.077 | -0.140 | 0.401 |
| GrimAA | -0.699 | **0.004** | -0.602 | **0.025** |
| DunedinPACE | -0.728 | **3.34×10^-12^** | -0.273 | **0.019** |
| **ln(HDL-C)** |  |  |  |  |
| HorvathAA | 0.002 | 0.134 | -0.002 | 0.240 |
| HannumAA | -0.001 | 0.298 | -0.004 | **0.044** |
| PhenoAA | -0.002 | **0.018** | -0.002 | 0.264 |
| GrimAA | -0.007 | **0.004** | -0.011 | **3.51×10^-4^** |
| DunedinPACE | -0.006 | **1.71×10^-9^** | -0.007 | **5.59×10^-9^** |
| **LDL-C** |  |  |  |  |
| HorvathAA | 0.165 | 0.121 | -0.100 | 0.521 |
| HannumAA | -0.197 | 0.120 | -0.209 | 0.378 |
| PhenoAA | -0.246 | **0.028** | -0.174 | 0.238 |
| GrimAA | -0.738 | **0.001** | -0.393 | 0.133 |
| DunedinPACE | -0.559 | **2.68×10^-9^** | -0.095 | 0.378 |
| **ln(TG)** |  |  |  |  |
| HorvathAA | 0.001 | 0.418 | 0.001 | 0.787 |
| HannumAA | 0.002 | 0.478 | 0.003 | 0.361 |
| PhenoAA | 0.004 | 0.050 | 0.005 | **0.025** |
| GrimAA | 0.013 | **5.86×10^-5^** | 0.010 | **0.016** |
| DunedinPACE | 0.005 | **1.60×10^-5^** | 0.006 | **4.42×10^-4^** |

TC, total cholesterol; HDL-C, high-density lipoprotein; LDL-C, low-density lipoprotein; TG, triglycerides; HorvathAA, HorvathAge acceleration; HannumAA, HannumAge acceleration; PhenoAA, PhenoAge acceleration; GrimAA, GrimAge acceleration

Model: blood lipid level = epigenetic age acceleration + age at methylation measurement + sex + race/ethnicity + lipid-lowering medication status + body mass index + smoking status + educational attainment (less than high school degree, high school degree or equivalent, college degree and above)

^a^Sample size for TC, HDL-C, LDL-C, and TG: 2529, 2489, 2531, and 2527.

^b^Sample size for TC, HDL-C, LDL-C, and TG: 1282, 1242, 1282, and 1279.

^c^β corresponds to the change in blood lipid level associated with a 1-unit increase in epigenetic age acceleration.

P-value < 0.05 in bold

**Supplemental Table 11.** Associations between epigenetic age acceleration and total cholesterol or LDL-C levels in participants by lipid-lowering medication use at the time of cholesterol measurement

|  | Using lipid-lowering medication^a^ | | Not using lipid-lowering medication^b^ | |
| --- | --- | --- | --- | --- |
|  | **β^c^** | **P** | **β^c^** | **P** |
| **TC** |  |  |  |  |
| HorvathAA | 0.108 | 0.532 | 0.224 | 0.151 |
| HannumAA | -0.226 | 0.238 | -0.304 | 0.072 |
| PhenoAA | -0.153 | 0.402 | -0.262 | 0.088 |
| GrimAA | -0.418 | 0.144 | -0.916 | **0.001** |
| DunedinPACE | -0.456 | **5.95×10^-5^** | -0.684 | **1.08×10^-9^** |
| **ln(HDL-C)** |  |  |  |  |
| HorvathAA | 8.28×10^-5^ | 0.952 | 0.001 | 0.395 |
| HannumAA | -0.004 | **0.013** | -4.46×10^-4^ | 0.800 |
| PhenoAA | -0.002 | 0.119 | -0.002 | 0.076 |
| GrimAA | -0.006 | **0.010** | -0.011 | **1.63×10^-4^** |
| DunedinPACE | -0.005 | **2.70×10^-7^** | -0.007 | **8.55×10^-11^** |
| **LDL-C** |  |  |  |  |
| HorvathAA | 0.029 | 0.841 | 0.108 | 0.381 |
| HannumAA | -0.050 | 0.767 | -0.349 | **0.018** |
| PhenoAA | -0.143 | 0.307 | -0.315 | **0.025** |
| GrimAA | -0.395 | 0.079 | -0.860 | **2.67×10^-4^** |
| DunedinPACE | -0.343 | **6.67×10^-4^** | -0.465 | **5.24×10^-6^** |
| **ln(TG)** |  |  |  |  |
| HorvathAA | 0.001 | 0.479 | 0.002 | 0.545 |
| HannumAA | 0.002 | 0.396 | 0.002 | 0.439 |
| PhenoAA | 0.004 | **0.044** | 0.004 | 0.081 |
| GrimAA | 0.007 | **0.019** | 0.016 | **3.70×10^-4^** |
| DunedinPACE | 0.005 | **7.10×10^-4^** | 0.006 | **3.45×10^-5^** |

TC, total cholesterol; HDL-C, high-density lipoprotein; LDL-C, low-density lipoprotein; TG, triglycerides; HorvathAA, HorvathAge acceleration; HannumAA, HannumAge acceleration; PhenoAA, PhenoAge acceleration; GrimAA, GrimAge acceleration

Model: blood lipid level = epigenetic age acceleration + age at methylation measurement + sex + race/ethnicity + fasting status + body mass index + smoking status + educational attainment (less than high school degree, high school degree or equivalent, college degree and above)

^a^Sample size for TC, HDL-C, LDL-C, and TG: 1868, 1821, 1869, and 1867.

^b^Sample size for TC, HDL-C, LDL-C, and TG: 1943, 1910, 1944, and 1939.

^c^β corresponds to the change in blood lipid level associated with a 1-unit increase in epigenetic age acceleration.

P-value < 0.05 in bold

**Supplemental Table 12**. Associations between epigenetic age acceleration and incident CVD (n=2,293; 205 cases)

|  | **Incident CVD events in 2020** | | | |
| --- | --- | --- | --- | --- |
| Epigenetic age accelration | **β** | **SE** | **OR**^a^ | **P** |
| HorvathAA | -0.009 | 0.012 | 0.991 | 0.476 |
| HannumAA | 0.018 | 0.018 | 1.018 | 0.343 |
| PhenoAA | 0.014 | 0.013 | 1.014 | 0.300 |
| GrimAA | 0.060 | 0.028 | 1.061 | **0.038** |
| DunedinPACE | 1.382 | 0.729 | 3.985 | 0.065 |

HorvathAA, HorvathAge acceleration; HannumAA, HannumAge acceleration; PhenoAA, PhenoAge acceleration; GrimAA, GrimAge acceleration; SE, standard error; OR, odds ratio

Model: log(odds of incident CVD) ~ epigenetic age acceleration + age at methylation measurement + sex + race/ethnicity + fasting status + lipid-lowering medication use + body mass index + smoking status + educational attainment (less than high school degree, high school degree or equivalent, college degree and above)

^a^OR corresponds to the increase in the odds of CVD associated with a 1-unit increase in the measure of epigenetic age acceleration.

P-value < 0.05 in bold


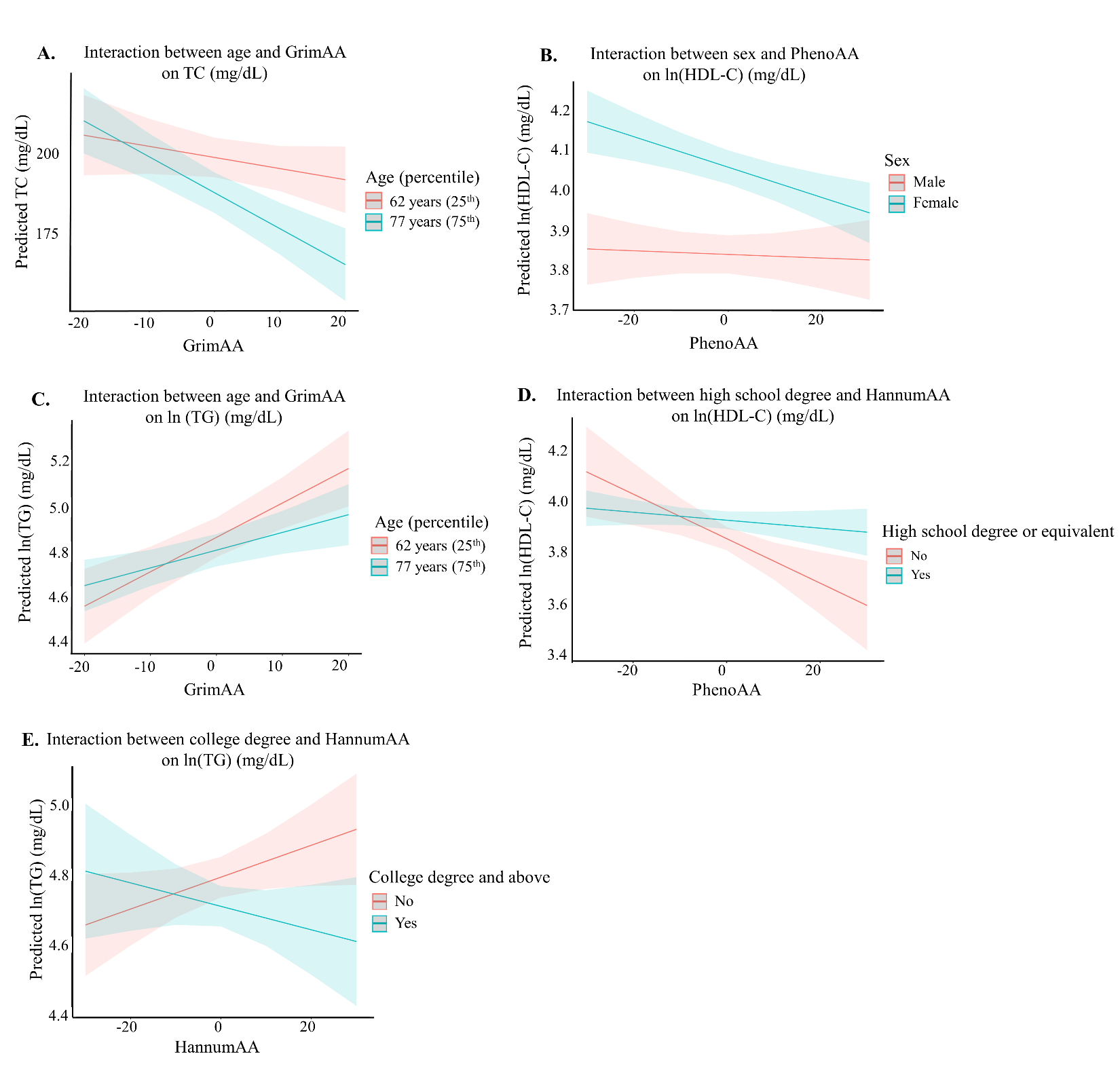


**Supplemental Figure 1.** Plots of predicted blood lipid levels by by GrimAA, PhenoAA, or HannumAA across demographic levels in the full sample (P_interaction_ < 0.05). **A**: predicted TC by GrimAA at the 25^th^ (62 years) and 75^th^ percentile (77 years) of age. **B**: predicted TG by GrimAA at the 25^th^ (62 years) and 75^th^ percentile (77 years) of age. **C**: predicted ln(HDL-C) by PhenoAA for males and females. **D**: Predicted ln(HDL-C) by HannumAA for participants with less than high school degree and participants with high school degree and above. **E**: Predicted ln(TG) by HannumAA for participants with less than college degree and participants with college degree and above.

TC, total cholesterol; HDL-C, high-density lipoprotein; TG, triglycerides; GrimAA, GrimAge acceleration; PhenoAA, PhenoAge acceleration; HannumAA, HannumAge acceleration

Interaction model: blood lipid level ~ epigenetic age acceleration + age at methylation measurement + sex + race/ethnicity + fasting status + lipid-lowering medication use + body mass index + smoking status + high school degree or equivalent + college degree and above + epigenetic age acceleration × demographic factor

Only interactions with GrimAA, PhenoAA, or HannumAA and demographic factors with P_interaction_ < 0.05 in the interaction model are shown.

Age was centered for the interaction analysis.

The line and corresponding confidence intervals represent the predicted blood lipid levels at the corresponding value of epigenetic age acceleration.
